# Si Wire Supported MnO_2_/Al/Fluorocarbon 3D Core/Shell Nanoenergetic Arrays with Long-Term Storage Stability

**DOI:** 10.1038/s41598-017-07148-1

**Published:** 2017-07-27

**Authors:** Ying Zhu, Xiang Zhou, Chun Wu, Hua Cheng, Zhouguang Lu, Kaili Zhang

**Affiliations:** 1 Department of Mechanical and Biomedical Engineering, City University of Hong Kong, 83 Tat Chee Avenue, Kowloon, Hong Kong; 2 National Special Superfine Powder Engineering Research Center of China, Nanjing University of Science and Technology, Nanjing, China; 3 Department of Materials Science and Engineering, Southern University of Science and Technology, Shenzhen, China

## Abstract

Three-dimensional MnO_2_/Al/fluorocarbon core/shell nanoenergetic arrays are prepared on silicon substrate that is with silicon wires on top. Silicon wires are first prepared as the scaffolds by maskless deep reactive ion etching of silicon wafer, which is followed by the hydrothermal growth of MnO_2_. Al and fluorocarbon are then deposited sequentially around the silicon wire (Si-W) supported MnO_2_ arrays by magnetron sputtering to realize the core/shell nanoenergetic composite. Several characterization techniques are used to investigate the prepared Si-W/MnO_2_/Al/fluorocarbon arrays, including the scanning electron microscopy, transmission electron microscopy, energy dispersive spectroscopy, X-ray photoelectron spectroscopy, and thermal analysis. 3D upright aligned core/shell structure with an intimate contact between MnO_2_ and Al is confirmed from the morphological characterization. Superhydrophobicity is achieved after the fluorocarbon coating. Most importantly, the Si-W/MnO_2_/Al/fluorocarbon nanoenergetic arrays show no decay of energy density after 9 months of storage, indicating potential applications in nanoenergetics-on-a-chip when long-term storage is needed.

## Introduction

Nanoenergetic materials refer to the energetic composites that are composed of fuels and oxidizers with nanoscale characteristics^1–3^. Compared with traditional energetic materials, including propellants, explosives, and pyrotechnics, nanoenergetic materials have received continuously increasing interest in recent years because of the unique performance in combustion^4–6^, ignition^7–9^, and energy release^10, 11^, leading to diverse promising applications in both military and civilian fields, such as gas generators, rapid initiation, micro-actuation, burn rate modifiers, synthesis and processing of materials, and biomedicine-related applications^12–17^. Recently, efforts are made to obtain nanostructured energetic composites, such as the reactive multilayered films^18–21^, porous substrate based materials^22–24^, and nanowires/rods based core/shell energetic materials^25–30^, in which the distribution and contact conditions of fuel and oxidizer are greatly improved for better performance^31^. The planar reactive multilayered films, which have received the most attention to date, are generally composed of at least two alternating thin layers of reactants that are fabricated by vapor deposition techniques^18^. The multilayered films can achieve a precisely controlled thickness, but the fabrication process requires a high-performance instrument and is time-consuming as a certain thickness requires dozens of modulation cycles for nanoscale multilayers. Porous substrate, such as porous silicon^22^ and 3D ordered macroporous membrane^23, 24^, can help to realize an enhanced reaction due to the more intimate contact between fuels and oxidizers either as a reactant itself or just as a frame. However, the low filling ratio of impregnated materials in both microscale and nanoscale pores remains an issue for porous silicon based nanoenergetics. As for 3D ordered macroporous membrane that has a simple fabrication process, the strength of the membrane, its adhesion to the substrate, and whether the deposited fuel can cover the bottom of a sufficiently thick membrane without affecting the structure is not clear.

Nanowires/rods based core/shell materials refers to the composites that one of the constituents, whether the oxidizer or the fuel, is entirely packed as the core in the form of nanowires or nanorods by the other shell constituent, in which the individual core/shell unit could be obviously identified^31^. The nanowires/rods based core/shell composites exhibit an enhanced reactivity and reliability resulting from the much more homogeneous and ordered distribution of oxidizers and fuels accompanied with increased contact area and intimacy, as well as the potential enhancement in long-term storage stability realized by oxide or hydrophobic shell^29, 30^. However, the nanowires prepared by the thermal oxidation method are not completely upright and leave a microscale oxide film beneath them, which has a negative effect on the energetic properties^28^. In addition, for the nanowires/rods that deposited or grown directly on the substrate, apart from the relative poor adhesion to the substrate, it is challenging to obtain an extreme long wire/rod.

**Figure 1:**
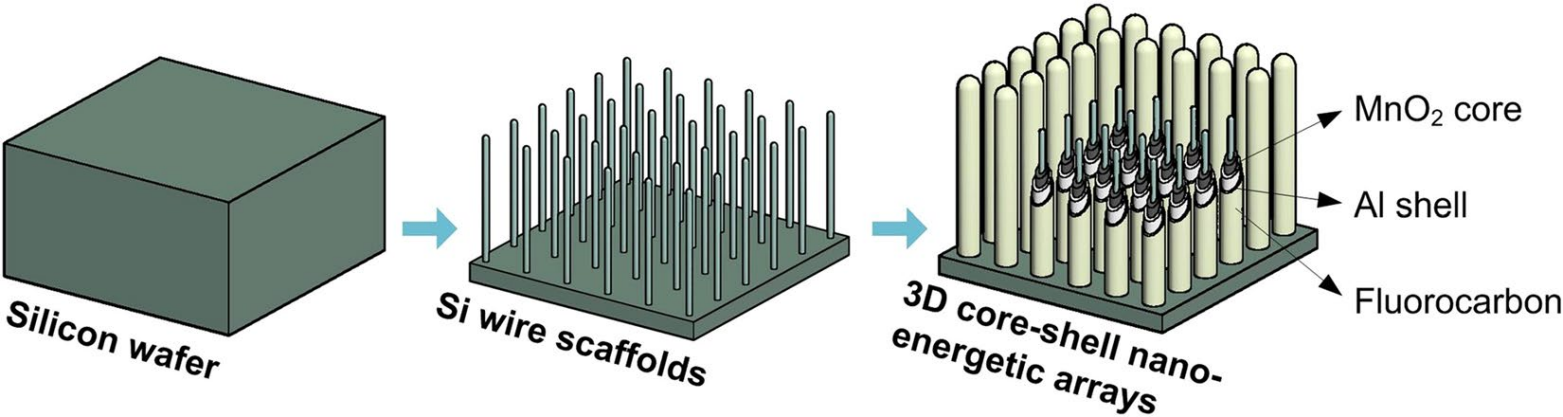
Schematic illustration of the fabrication process of the 3D silicon wire supported MnO_2_/Al/fluorocarbon nanoenergetic arrays.

Herein, we present the silicon wire (Si-W) supported MnO_2_/Al/fluorocarbon core/shell nanoenergetic composite. Unlike the simple nanowires/rods core/shell structure described above, the silicon wire arrays fabricated by deep reactive ion etching of silicon wafer are used as the scaffolds for the growth of MnO_2_ layer and subsequent deposition of aluminum and fluorocarbon, and together form a 3D nanoenergetic material. The design of the silicon wires as the scaffold has the following advantages. First, the silicon wires are all well-aligned regular upright arrays, which can facilitate the growth and/or deposition of later constituents. Second, the length of the core/shell structure can be easily adjusted by altering the length of the silicon wire scaffolds, and longer wires/rods based core/shell nanoenergetic materials is possible to be obtained, adding the spatial dimension to a finite unit area and thus increasing the specific surface area for fuels and oxidizers as well as the amount of reactant. Moreover, the silicon wires and the beneath silicon substrate are inherently integrated, which not only makes the prepared energetic material without any adhesion issue, but also facilitates the integration with microelectromechanical systems (MEMS). In addition, the silicon wire scaffold design is universal and can be extended to other oxidizer-fuel system to integrate a variety of nanothermite agents. The schematic of the fabrication process is shown in Fig. 1. After a simple and effective deposition of fluorocarbon as well studied in our previous work^30, 32, 33^, the ultra-long upright arrays together with the nano-textured surface that benefits from the aluminum particles and beneath porous MnO_2_ nanosheets on each array, make the realized energetic composite superhydrophobic, thus ensuring the long-term storage stability.

## Results and Discussion

### Morphological and Compositional Analysis

**Figure 2:**
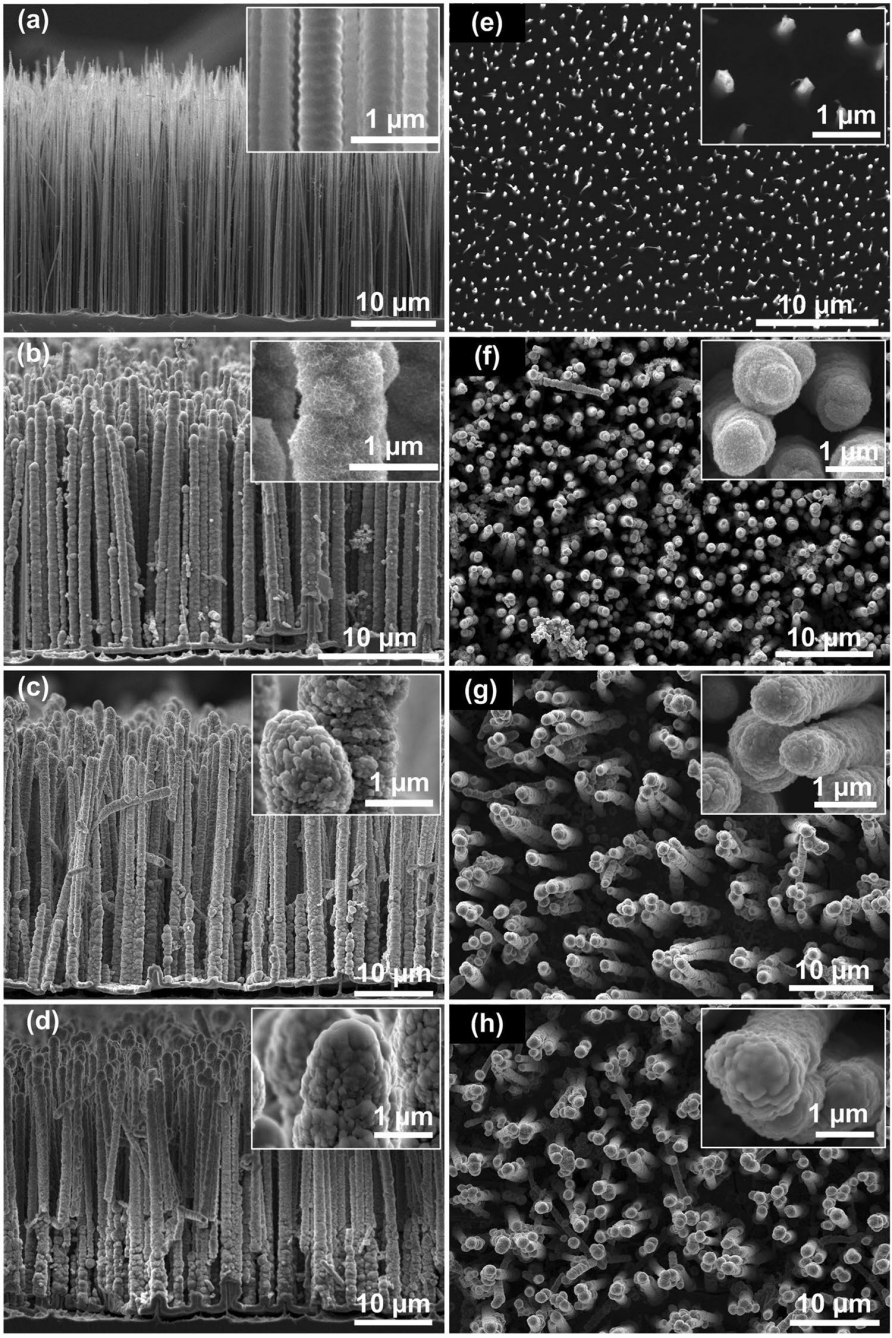
Cross-sectional and top-view SEM images of (**a** and **e**) Si-W arrays, (**b** and **f**) Si-W/MnO_2_ rods, (**c** and **g**) Si-W/MnO_2_/Al nanoenergetic arrays, and (**d** and **h**) Si-W/MnO_2_/Al/fluorocarbon arrays. Insets are respective magnified images.

Figure 2 shows the cross-sectional (Fig. 2a–d) and top-view (Fig. 2e–h) SEM images of Si wire arrays, Si-W/MnO_2_ rods, Si-W/MnO_2_/Al nanoenergetic arrays and Si-W/MnO_2_/Al/fluorocarbon arrays, respectively. Insets are magnified images of respective materials. As shown in Fig. 2a, the Si wire arrays are orderly upright, and the average length of Si wires is ~26 µm with a diameter in submicron range. The magnified inset image shows the special helix-like pattern of Si wires caused by alternating passivation and etching of DRIE process. Only scattered dots can be seen from the top-view image shown in Fig. 2b, and individual Si wire is irregular with dimension in nanometer range as shown in the magnified inset image. After hydrothermal process, the arrays remain upright, and an obvious increase in diameter of each wire can be seen in Fig. 2b and f, as a layer of MnO_2_ forms outside Si-W arrays and makes the wires grow to be rod-like. The surface of Si wafer without Si-W arrays is also coated with a layer of MnO_2_ due to the conformity of hydrothermal method. The thickness of MnO_2_ layer is about 250 nm, with a porous structure similar to a dense loofah sponge that consists of a lot of nanosheets as shown in inset of Fig. 2b.

The deposition rate of Al or fluorocarbon by RF magnetron sputtering on rods is difficult to determine accurately as it is different from that on a flat substrate surface. Thus, a nominal thickness according to the deposition rate on a flat silicon surface is used to describe the theoretic thickness of Al or fluorocarbon. Figure 2c and g show the arrays after Al deposition with a nominal thickness of 1 µm. The diameter of each rod increases slightly, and the loofah sponge-like structure is covered by Al nanoparticles that form tiny embedded lumps. The intimate interfacial contact between Al and MnO_2_ can enhance the mass transport thus improve the reaction velocity in solid state reaction^31^. After the deposition of fluorocarbon with a nominal thickness of 120 nm, the surface of rods becomes faint hazy as shown in inset images of Fig. 2d and h. The fluorocarbon layer of Si-W/MnO_2_/Al/fluorocarbon energetic rods is thinner at the bottom, as the brightness differs from the top part as shown in Fig. 2d. It is possibly due to the limited deposition time, overlong pathway and/or relatively crowded rods, though the ejected particles in magnetron sputtering process have relatively high kinetic energies. However, as discussed later, it is enough to get an excellent performance for Si-W/MnO_2_/Al/fluorocarbon energetic composites. Besides, a uniform covering effect is possible to obtain by adjusting related parameters as demonstrated in our previous investigation^33^.

**Figure 3:**
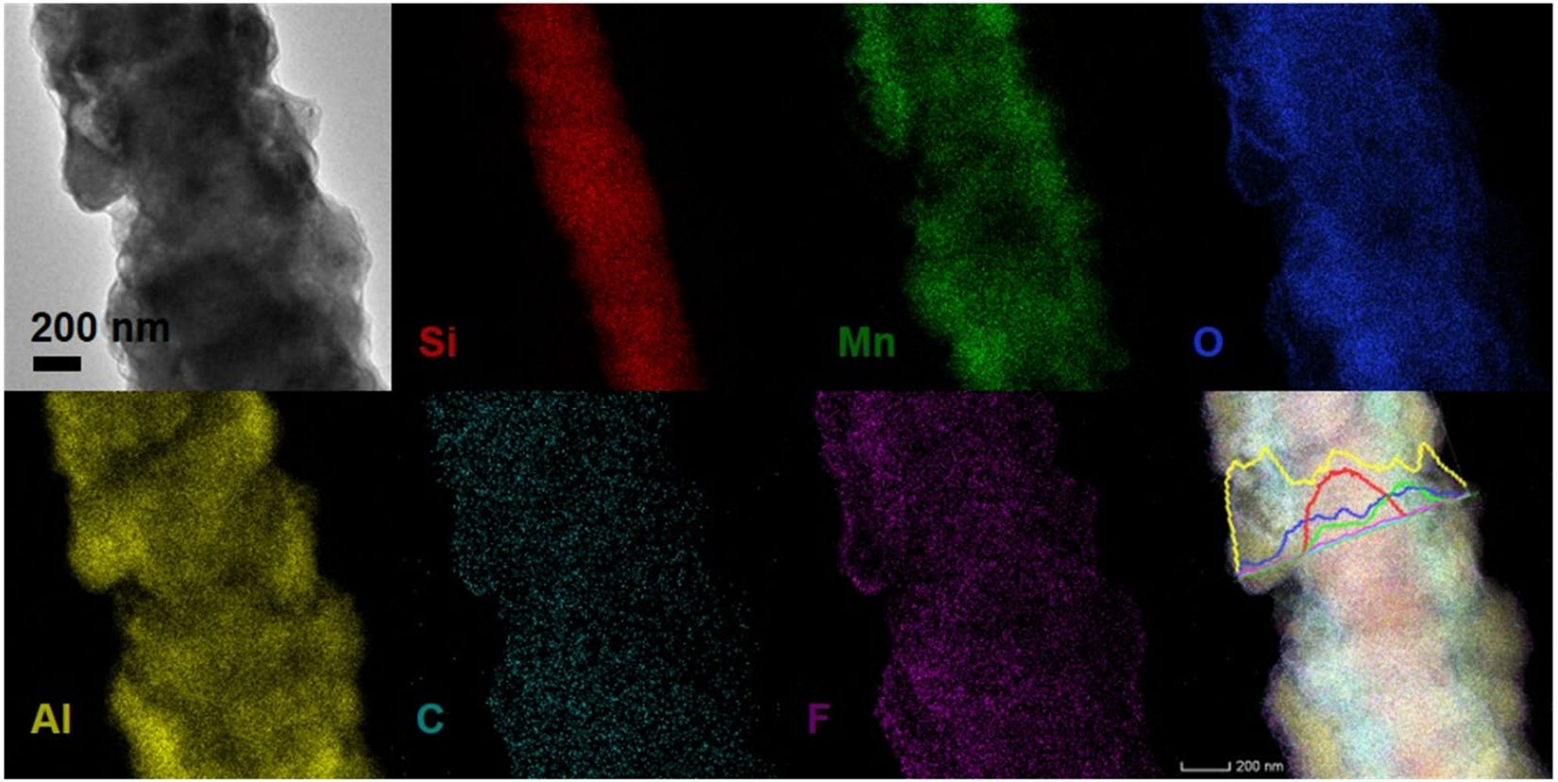
TEM elemental mapping images and line scan spectra of the Si-W/MnO_2_/Al/fluorocarbon rod.

TEM elemental mapping of individual Si-W/MnO_2_/Al/fluorocarbon rod is conducted to further confirm the core-shell structure. The sample is a Si-W/MnO_2_/Al/fluorocarbon rod with 1 μm Al and subsequent 120 nm fluorocarbon deposition. As shown in Fig. 3, Si signals are obviously dominant in the scaffold region, where the Mn and O elements are uniformly distributed over. Al particles wrap the inner rods core and achieve a good coating effect, which are covered by the C and F elements from the subsequently deposited fluorocarbon, forming the MnO_2_/Al/fluorocarbon core/shell structure. The elemental distribution of Si, Mn, O, Al, C and F is further analyzed by TEM line scan. The Si signals are only dominant in the scaffold region, whereas the Mn and Al signals are strong across the outer region. Further characterizations of the Si-W/MnO_2_/Al/fluorocarbon rod are carried out by high-resolution TEM (HRTEM) and selected area electron diffraction (SAED) as shown in Supplementary Fig. S1. The boundaries of amorphous fluorocarbon and aluminum area can be clearly seen in the HRTEM image as shown Supplementary Fig. S1b, where the fluorocarbon has a thickness of about 15 nm. As shown in Supplementary Fig. S1c, the lattice fringes with an interplanar spacing of 0.239 nm could be identified as the (111) plane of Al, and the inset SAED pattern shows its polycrystalline feature. The aforementioned characterizations confirm the successful preparation of Si wire supported MnO_2_/Al/fluorocarbon core/shell structure, which can be further proved by the following characterizations.

**Figure 4:**
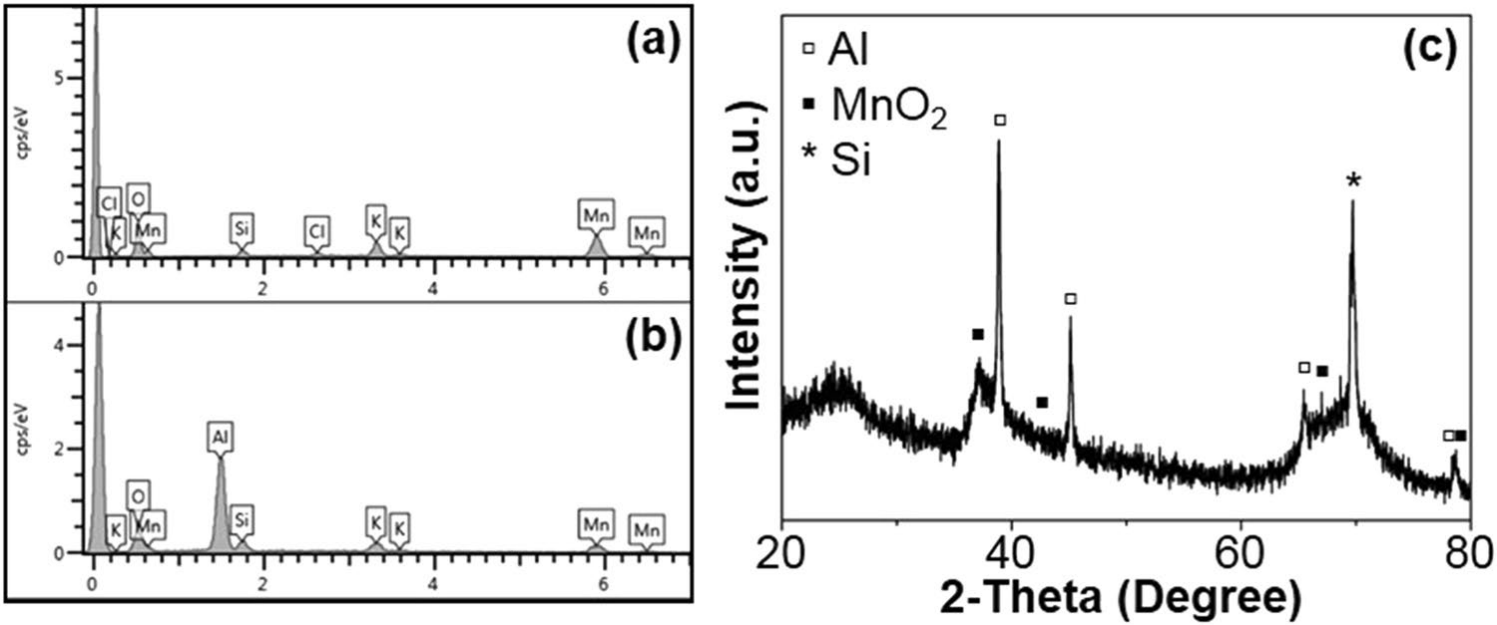
EDS patterns of (**a**) Si-W/MnO_2_ and (**b**) Si-W/MnO_2_/Al, and (**c**) XRD pattern of Si-W/MnO_2_/Al.

Figure 4 shows the EDS patterns of Si-W/MnO_2_ and Si-W/MnO_2_/Al samples and the XRD pattern of Si-W/MnO_2_/Al composite. The elements of Mn and O are detected from the Si-W/MnO_2_ sample as shown in Fig. 4a. Si signal that can be attributed to Si substrate and/or Si wires is found due to the large detection depth of EDS. Elements of Cl and K can also be found in the pattern, which possibly originate from contamination. As for Si-W/MnO_2_/Al sample, a relatively strong Al signal appears as shown in Fig. 4b, which could be attributed to the deposited aluminum shell. Similarly, K signals due to contamination and Si signal that originates from silicon substrate and/or supported silicon wires appear in the pattern. Correspondingly, XRD pattern of Si-W/MnO_2_/Al as shown in Fig. 4c is indexed to Al and MnO_2_ corresponding to Al (JCPDS 04-0787) and MnO_2_ (JCPDS 30-0820), respectively. The peaks located at 37.5°, 44.7°, 65.1°, and 78.2° correspond to the (111), (200), (220), and (311) planes of Al, respectively. The diffraction peaks at 37.1° and 78.9° represent the characteristic reflections of (100) and (200) of MnO_2_, respectively. Also, a diffraction peak at 69.2° caused by the (400) lattice plane of silicon is observed. The results verify the successful growth of MnO_2_ as well as the subsequent deposition of Al. The diffraction intensity of MnO_2_ peaks is relatively weak due to its low crystallinity.

**Figure 5:**
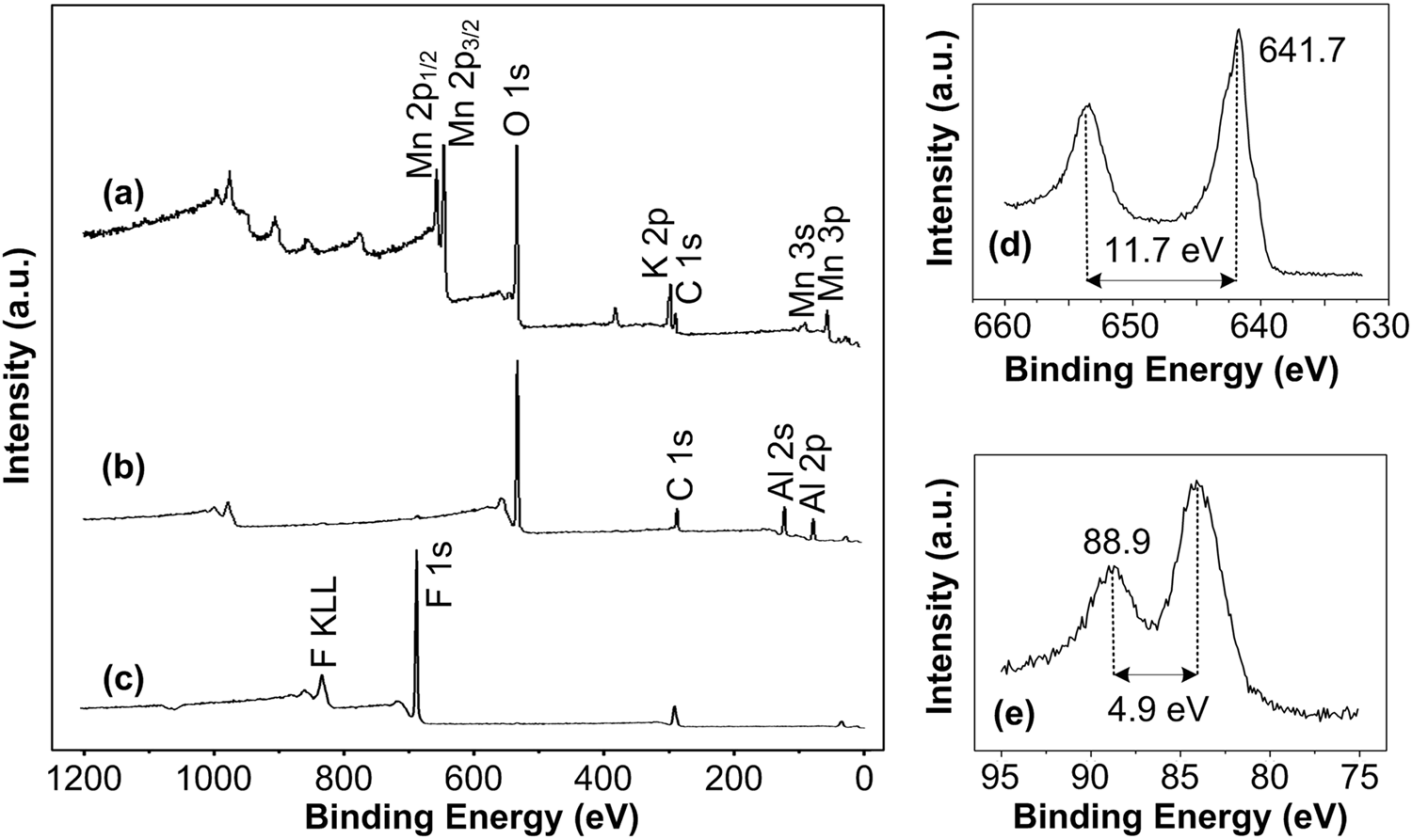
XPS survey spectra of (**a**) Si-W/MnO_2_, (**b**) Si-W/MnO_2_/Al, and (**c**) Si-W/MnO_2_/Al/fluorocarbon arrays, and high-resolution spectra of (**d**) Mn 2p and (**e**) Mn 3 s peaks.

XPS is applied to determine the composition of the arrays. Figure 5 shows the XPS spectra of the Si-W/MnO_2_, Si-W/MnO_2_/Al, and Si-W/MnO_2_/Al/fluorocarbon arrays. The survey spectrum of Si-W/MnO_2_ is shown in Fig. 5a, which illustrates the existence of manganese by peaks assigned to Mn 2p_1/2_, Mn 2p_3/2_, Mn 3 s and Mn 3p. Figure 5d shows a high-resolution spectrum of Mn 2p, where the binding energy of Mn 2p_3/2_ (641.7 eV) and a spin energy separation of 11.7 eV between Mn 2p_1/2_ and Mn 2p_3/2_ peaks are consistent with those in MnO_2_^34^. Besides, a peak energy separation of 4.9 eV is observed from the high-resolution spectrum of Mn 3 s as shown in Fig. 5e, which is in agreement with the manganese oxidation state at 4^35^, indicating that MnO_2_ layer is successfully formed on the surface of Si wire arrays because of the decomposition of KMnO_4_ in water as^36^

$$4KMn{O}_{4}+2{H}_{2}O=4Mn{O}_{2}\downarrow +4KOH+3{O}_{2}\uparrow .$$

Moreover, Mn peaks disappear in the survey spectrum of Si-W/MnO_2_/Al as shown in Fig. 5b, and instead, Al 2 s and Al 2p peaks show up, demonstrating Si-W/MnO_2_ arrays are fully covered by the deposited Al layer at the surface. The high-resolution XPS spectrum of Al 2p shown in Supplementary Fig. S2 demonstrates the existence of Al and alumina, where the oxide can be attributed to the natural oxidation in air before testing. Supplementary Fig. S3 shows the XPS survey scan spectrum of the Si wire arrays and the high-resolution spectra of C 1 s and Si 2p. Figure 5c shows the survey scan XPS spectrum of Si-W/MnO_2_/Al/fluorocarbon. The C 1 s, F 1 s, and F KLL Auger electron signals indicate the main elements within the outer layer are C and F. The C 1 s high-resolution XPS spectrum of the fluorocarbon thin film shown in Supplementary Fig. S4 demonstrates the existence of C-F_3_ (293.1 eV), C-F_2_ (291.3 eV), C-F (289.3 eV), and C-CF_n_ (287.1 eV)^37, 38^, with a calculated concentration percentage of 30.9%, 33.5%, 21.6%, and 14.0%, respectively. Beside the lowest surface free energy provided by CF_3_ groups that could result in a hydrophobic or even superhydrophobic surface^39^, the higher concentrations of CF_2_ and CF_3_ can be beneficial for energetic applications from the chemical reaction point of view^32^.

### Wettability Test

**Figure 6:**
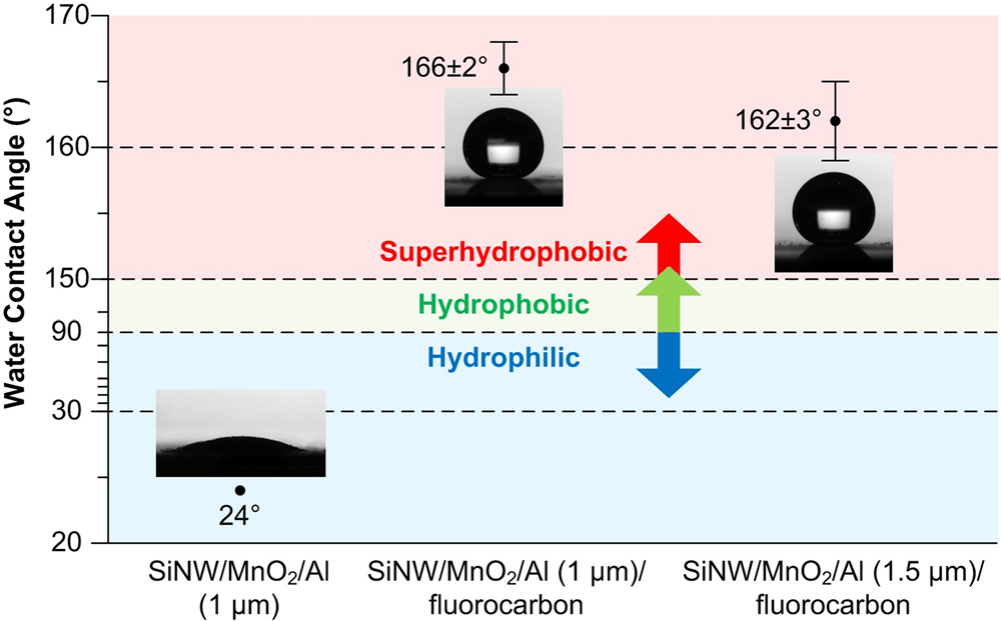
Water contact angles of different samples.

Figure 6 shows water contact angle data for Si-W/MnO_2_/Al and Si-W/MnO_2_/Al/fluorocarbon with different nominal thickness of Al. Water contact angle of Si-W/MnO_2_/Al with nominal 1 µm Al is about 24°, demonstrating the hydrophilicity of this composite. After being deposited with nominal 120 nm fluorocarbon, Si-W/MnO_2_/Al/fluorocarbon becomes superhydrophobic, as the water drop contact angle of samples with nominal 1 µm and 1.5 µm Al are about 166° and 162°, respectively. According to our previous work^32, 33^, surface energy of nanoenergetic composites with nano-textures decreases after being coated with fluorocarbon. Besides, with surface texture that has a comparatively high aspect ratio can constitute a superhydrophobic surface as conforming to the Cassie-Baxter model^40, 41^. The slightly decrease in hydrophobicity for samples with thicker Al as shown in Fig. 6 may be due to the smoothing effect while more Al particles are deposited onto the surface. Overall, the thickness of aluminum exerts little influence on composites’ superhydrophobicity.

### Thermal Analysis

**Figure 7:**
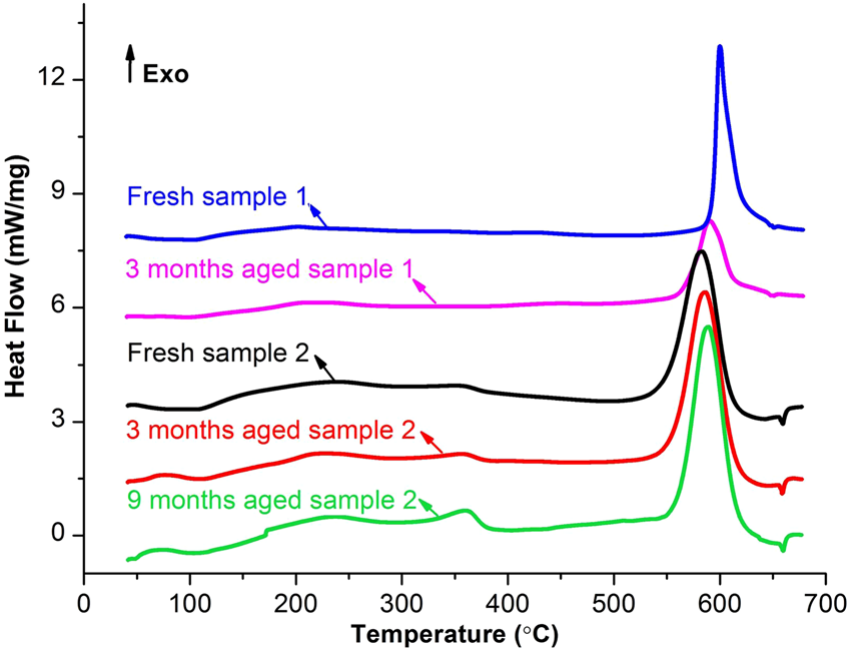
DSC curves of freshly prepared and aged samples of Si-W/MnO_2_/Al (sample 1) and Si-W/MnO_2_/Al/fluorocarbon (sample 2).

Figure 7 shows the heat flow curves of Si-W/MnO_2_/Al (Sample 1) and Si-W/MnO_2_/Al/fluorocarbon (Sample 2), both including freshly prepared and aged ones. Curves are shifted arbitrarily in y-axis for clarity. The nominal thickness of deposited aluminum is 1 µm, and the value for fluorocarbon is 120 nm for sample 2. All curves give a similar trend with only one main exothermic peak. The main exothermic peaks of all samples are before 600 °C and can be attributed to the thermite reaction between MnO_2_ and Al as mentioned above. It is a specific evidence to confirm the solid state reaction between MnO_2_ and Al in this core-shell structure, as the reaction happens before Al melts (660 °C), which is great potential for practical use in nanoenergetics-on-a-chip. Specifically, the onset reaction temperature of main exothermic peak of fresh sample 1 is 592 °C, while this value is 550 °C for fresh sample 2. The difference in onset reaction temperature between them could be attributed to the fluorocarbon layer. As shown in Fig. 7, apart from the main exothermic peak, another smaller exotherm appears on each of the three curves of sample 2 around 320 °C. It presumably originates from the fluorination of the Al/Al_2_O_3_ after the low-temperature decomposition of fluorocarbon^30, 33^. Reports have shown that the pre-ignition reaction observed in various fluoropolymers-containing formulations could intensify the over-all exothermic reaction and combustion process^42–45^. Besides, a recent research has stated that the pre-ignition reaction between Al_2_O_3_ and fluorine-containing polymer can affect the heat-release characteristics^46^. In this study, similarly, fluorocarbon could possess a pre-ignition reaction with Al/Al_2_O_3_ after its decomposition at a relatively low temperature, which may increase the local temperature and thus trigger the interfacial reaction between Al and MnO_2_, resulting in a lower onset reaction temperature. Due to the limitation of Q20 in working temperature, differential thermal analysis (DTA, TA Instruments Q600) is used to determine the heat release property above 680 °C as a complement. An unobvious exothermic signal appears over 800 °C as shown in Supplementary Fig. S5, which is negligible compared to the main exothermic reaction before 660 °C.

Experimental data of heat release are tabulated in Table 1. For freshly prepared Si-W/MnO_2_/Al and Si-W/MnO_2_/Al/fluorocarbon, the determined heat of reaction is about 1071 J/g and 1008 J/g, respectively. It is noticed that the measured heat of reaction is only around 25% of the theoretical one (4178 J/g)^47^. This difference can be attributed to the dead weight of silicon wires that act as the scaffold of energetic MnO_2_/Al and MnO_2_/Al/fluorocarbon materials. Silicon wires are scraped off from the substrate wafer together with the outer reactive layers, and they are not involved in reaction during heating process as silicon is not very chemically reactive unless a high temperature is achieved^33^.

**Table 1:** Heat of thermite reaction of Si-W/MnO_2_/Al and Si-W/MnO_2_/Al/fluorocarbon.

	Heat of thermite reaction (J/g)	
	Si-W/MnO_2_/Al	Si-W/MnO_2_/Al/fluorocarbon
Freshly prepared	1071	1008
3 months aged	845	1105
9 months aged	—	1134

After being stored for 3 months in a dry cabinet that maintains 50% relative humidity at 25 °C, the heat of reaction of sample 1 decreases to 845 J/g, which is a 21% decrease in energy density. While for samples 2, the determined heats of reaction for aged 3 and 9 months’ ones are 1105 and 1134 J/g, respectively, demonstrating no decay in energy density during storage. The slight increase of values may due to small variation between each batch of the fabricated samples. Actually, the Si wire scaffolds are of great benefit to the MnO_2_/Al/fluorocarbon nanoenergetic composite. The Si wire scaffolds make the core-shell structure three-dimensional, which provides a much higher specific surface area that could increase the contact area between fuel and oxidizer and improve the solid-state reaction. Furthermore, the high aspect ratio rod arrays do help to form the superhydrophobic surface. The superhydrophobic surface consisting of CF_n_ groups can repel water molecules, which is believed to provide an improved protection effect for aluminum in changeable environment and ensure the long-term storage stability^30^. In addition, an enhanced protective effect benefits from rod-like core/shell compared with a rather planar textured structure. The sandwich structured CuO/Mg/fluorocarbon exhibits a 18% loss in energy density after being exposed in 95% relative humidity at 35 °C for 10 days^32^. Whereas the 3D core/shell Si-W/MnO_2_/Al/fluorocarbon rod arrays in this study maintain the energy density well over 9 months. Similarly, the rod-like Mg/CuO core/shell energetic materials show no decay in energy density after 1 month’s storage^29^. Although the parameters of storage are not the same, the rod-like core/shell structure exhibits a distinct superiority in storage stability over a much longer period of time. Besides, as originating from the silicon substrate, the Si wire scaffolds provide a tightly bonding interface for integrating nanoenergetic materials with MEMS, demonstrating a potential application in functional nanoenergetics-on-a-chip.

### Summary

3D superhydrophobic MnO_2_/Al/fluorocarbon nanoenergetic arrays supported by silicon wires are prepared and investigated in this study. It is found that MnO_2_ core grows around the orderly Si wires uniformly, and the later deposited Al shell layer acquires the nano-texture from the underlying Si-W/MnO_2_ arrays, thus forming a superhydrophobic surface after fluorocarbon deposition. Besides the increased specific surface area, the superhydrophobicity enables the Si-W/MnO_2_/Al/fluorocarbon to preserve the chemical energy well after long-term storage, as the nanoenergetic composite shows no decay of energy density even after 9 months of storage. Despite the dead weight of silicon wire scaffolds, the heat of reaction is still promising. The results indicate that the new 3D nanoenergetic material has potential applications in nanoenergetics-on-a-chip, especially when long-term storage is needed. The concept adopted here may also be applied to other fuels (e.g. Mg) and oxidizers (e.g. Co_3_O_4_, Fe_2_O_3_, and CuO) to achieve different promising nanoenergetic arrays with long-term storage stability.

## Methods

### Fabrication of Si-W/MnO_2_/Al/fluorocarbon arrays

Silicon wafer substrate with upright Si wire arrays is fabricated by a mask-free deep reactive ion etching (DRIE) method^48^. The polished silicon wafer (4 inch in diameter and 500 µm in thickness) is processed through a 6 s/6 s passivation/etching cycle with total processing time of 48 min. The details of DRIE process can be found in our previous work^33^. The MnO_2_ coating is prepared via a simple hydrothermal process. The wafer with Si wire arrays is treated with oxygen plasma to make it hydrophilic. Then the wafer is immersed into 0.01 M KMnO_4_ (AR, ≥99.5%) solution in a 100 mL Teflon-lined stainless steel autoclave, and the autoclave is subsequently maintained at 150 °C for 1.5 h. After cooling down to room temperature naturally, the wafer is taken out, washed with deionized water and ethanol several times, and dried at 60 °C overnight. Al and fluorocarbon are then deposited around the as-prepared Si-W/MnO_2_ arrays by RF magnetron sputtering (JunSun MGS-600 Sputtering System), with a sputtering power of 120 and 140 W, respectively. The targets are Al and PTFE, which are both 2 inch in diameter and 0.125 inch in thickness. The base vacuum and working pressure are 2 × 10^−6^ and 1.8 × 10^−3^ Torr, respectively. Argon gas acts as the working atmosphere with a flow rate of 30 sccm.

### Morphological and compositional characterization

The morphological and microstructural information of as-prepared samples are directly observed with a field-emission scanning electron microscope (FESEM, FEI Quanta 450) and a transmission electron microscope (TEM, FEI Tecnai G2 20). Energy dispersive spectroscopy (EDS, Oxford Instruments/INCA Energy 200) and X-ray photoelectron spectroscopy (XPS, Physical Electronics PHI5802) are used to get the structural and compositional information of as-prepared samples. The XPS measurement is carried out with a monochromatized Al Kα X-ray source (1486.6 eV), and the chamber pressure is on the order of 10^−9^ Torr during tests. No Ar etching is used before characterization.

### Wettability test and thermal analysis

The water drop contact angles of Si-W/MnO_2_/Al and Si-W/MnO_2_/Al/fluorocarbon surfaces are measured by KRÜSS DSA100 drop shape analysis system. A deionized water droplet with a volume of 5 µL is used each time. The exothermic reactions are characterized by differential scanning calorimetry (DSC, TA Instruments Q20). In order to prove the long-term storage stability of as-prepared 3D core/shell energetic composites, the freshly prepared sample and aged ones are compared by DSC. The aged samples are those stored in a dry cabinet that maintains 50% relative humidity at 25 °C for 3 and 9 months, respectively. The samples are scraped off the silicon wafer for testing. DSC analyses are performed from 40 to 680 °C with a heating rate of 5 °C/min under 50 mL/min Ar flow. For all tests, Ar flow is started 30 min before temperature ramps up to replace the air in cell as much as possible. DSC data is analyzed by Universal Analysis 2000 software.

### Data availability

No datasets were generated or analyzed during the current study.

## Electronic supplementary material

Supplementary Information
