# Supplementary material for: Si Wire Supported MnO2/Al/Fluorocarbon 3D Core/Shell Nanoenergetic Arrays with Long-Term Storage Stability
Source: Sci Rep. 2017 Jul 27;7:6678. doi: 10.1038/s41598-017-07148-1 (PMC5532262; doi:10.1038/s41598-017-07148-1)
Supplement: Supplementary file 1 — Supplementary Information [file 41598_2017_7148_MOESM1_ESM.pdf]

Supplementary Information

Si Wire Supported MnO_2/Al/Fluorocarbon 3D Core/Shell Nanoenergetic Arrays with Long-Term Storage Stability

Ying Zhu[a], Xiang Zhou[b], Chun Wu[a], Hua Cheng[c], Zhouguang Lu[c], and Kaili Zhang[*a]*

[a] Department of Mechanical and Biomedical Engineering, City University of Hong Kong, 83 Tat Chee Avenue, Kowloon, Hong Kong

[b] National Special Superfine Powder Engineering Research Center of China, Nanjing University of Science and Technology, Nanjing, China

[c] Department of Materials Science and Engineering, Southern University of Science and Technology, Shenzhen, China

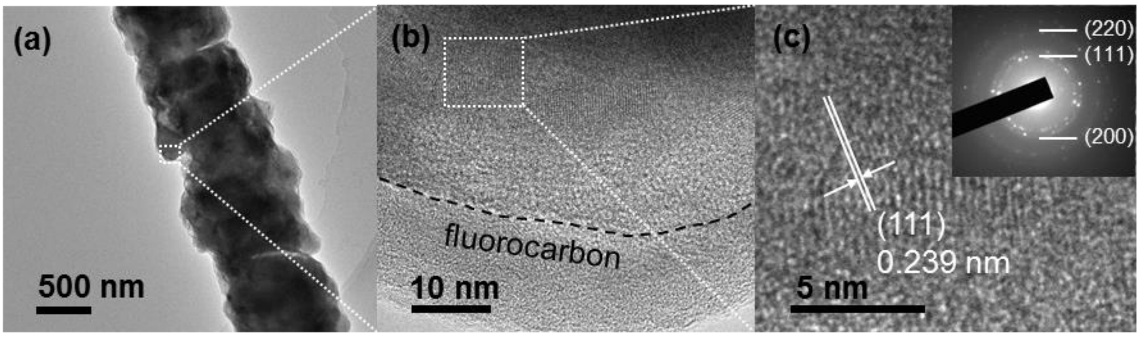

Supplementary Figure S1. (a) Low-magnification TEM image of core/shell structured Si-W/MnO_2/Al/fluorocarbon rod, and (b-c) HRTEM images of the white dashed frame part. Inset is the SAED pattern of a section of sample.

The rings shown in inset of Supplementary Fig. S1c demonstrates the polycrystalline feature of outer Al layer, where the calculated *d* values of 0.24, 0.21, and 0.15 nm correspond to the (111), (200), and (220) Miller indices, respectively. As seen from the low-magnification TEM image of core/shell sample, the outer layer of aluminum is too thick to obtain a HRTEM of the MnO_2 area that encapsulated therein.

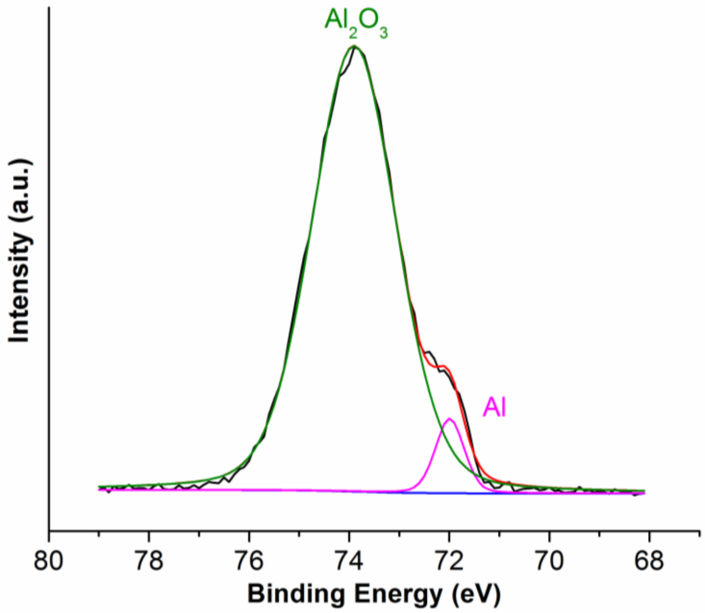

Supplementary Figure S2. Al 2p high resolution XPS spectrum of Si-W/MnO_2/Al.

The Al 2p spectrum contains two peaks as revealed by the curve fitting shown in Supplementary Fig. S2. The peak located at binding energy of 72.0 eV belongs to the metallic signal of aluminum[1]. The other peak at binding energy of 73.9 eV corresponds to the alumina[1], which forms naturally in air before testing.

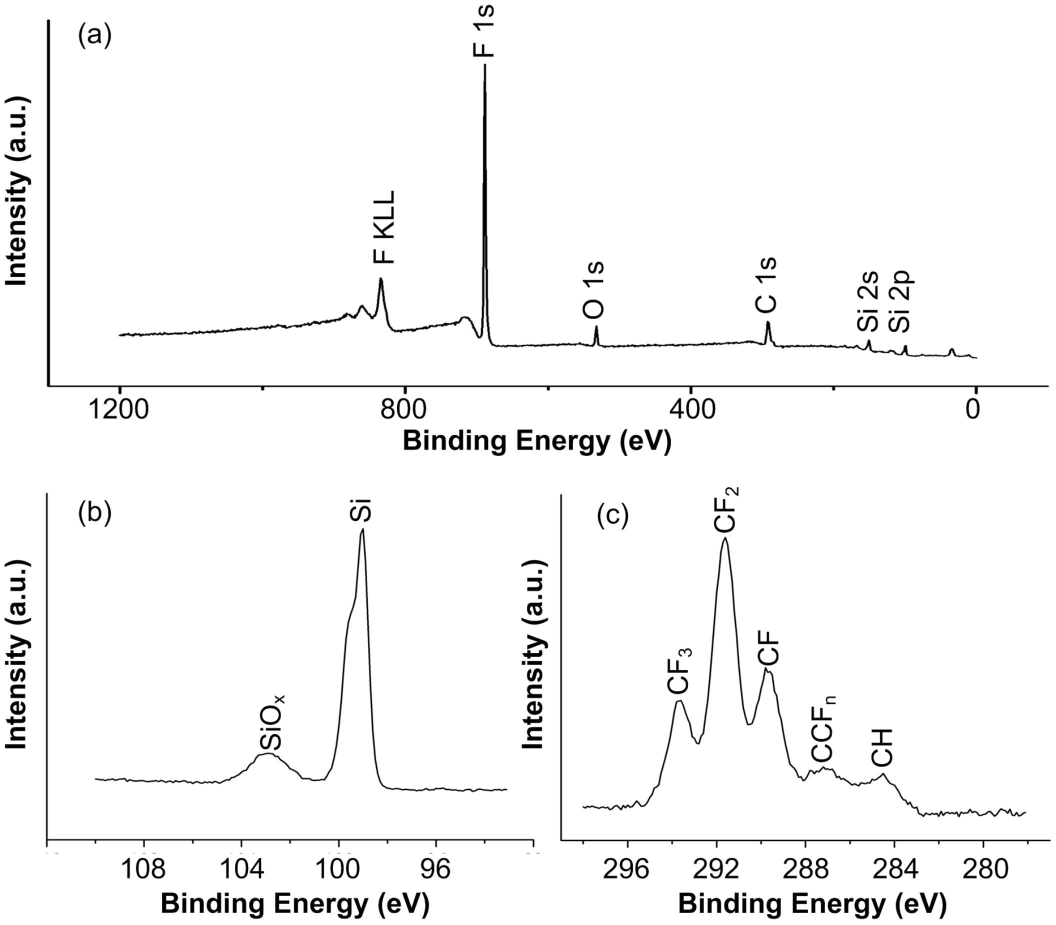

Supplementary Figure S3. (a) Survey scan XPS spectrum of Si wires and (b and c) high resolution spectra of Si 2p (b) and C 1s (a).

The XPS spectra of the Si wire arrays are similar to our previous results[2]. Signals of C, F, and Si are obvious in the survey scan spectrum as shown in Supplementary Fig. S3a, indicating the Si wires are coated with a fluorocarbon layer that forms during the passivation/etching cycle by C_4F_8 and SF_8-O_2, respectively. Supplementary Fig. S3b shows the high resolution

spectrum of Si 2p, in which Si oxide is found and causes the chemical shift. Supplementary Fig. S3c shows the high resolution spectra of C 1s, which indicates the fluorocarbon layer is consisted of CF_3, CF_2, CF, CCF_n, and CH functional groups[3,4].

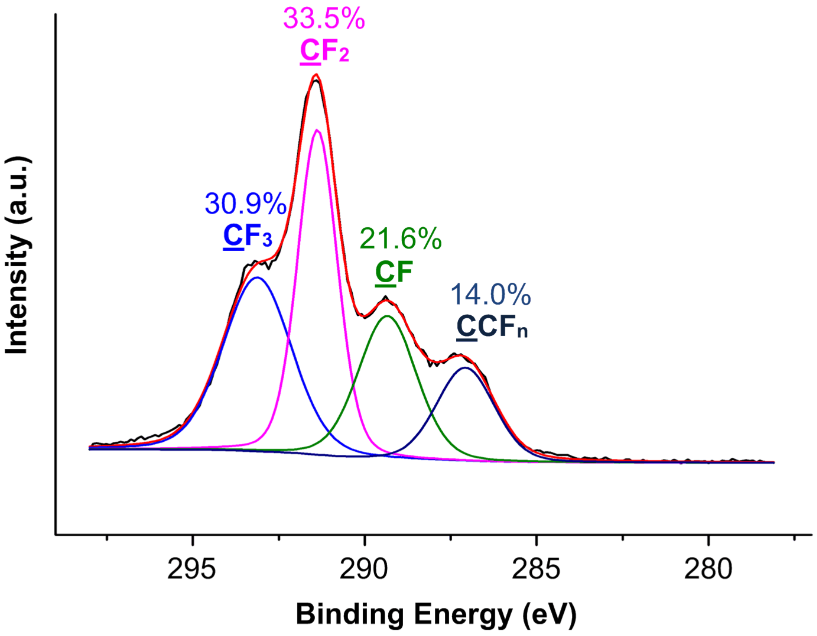

Supplementary Figure S4. C 1s high resolution XPS spectrum of Si-W/MnO_2/Al/fluorocarbon.

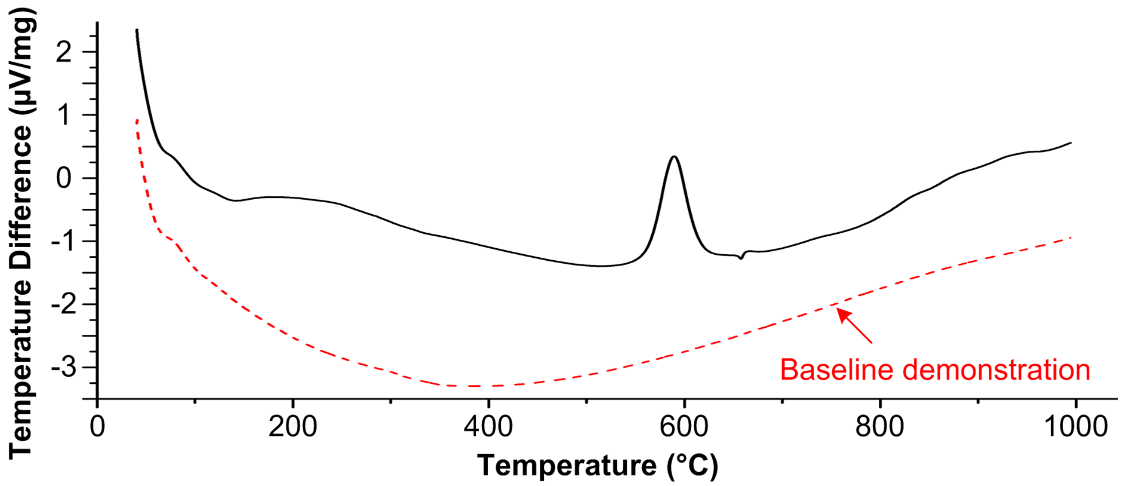

Supplementary Figure S5. DTA curve of fresh Si-W/MnO_2/Al/fluorocarbon.

DTA test is conducted from 40 ˚C to 1000 ˚C with a heating rate of 10 ˚C/min under an Ar flow at 100 mL/min. The Ar gas is started 30 min before the temperature increasing to remove the air within the chamber. The data is analyzed by Universal Analysis 2000 software. The baseline is not flat along with the temperature due to unknown instrumental issues; thus a red dotted line is drawn in the figure as the baseline demonstration. The baseline measurement is run with no sample in pan under the same program.

References

1. Moulder, J. F., Chastain, J. & King, R. C. *Handbook of X-ray photoelectron spectroscopy: a reference book of standard spectra for identification and interpretation of XPS data*. (Perkin-Elmer Eden Prairie, MN, 1992).
2. Zhou, X., Zhu, Y., Zhang, K., Lu, J. & Jiang, W. An extremely superhydrophobic and intrinsically stable Si/fluorocarbon energetic composite based on upright nano/submicron-sized Si wire arrays. *RSC Adv.* **5,** 106098–106106 (2015).
3. Cheng, Z. *et al.* Super-hydrophobic surface with switchable adhesion responsive to both temperature and pH. *Soft Matter* **8,** 9635 (2012).
4. Zhang, Y. *et al.* Deposition of fluoropolymer films on Si(100) surfaces by RF magnetron sputtering of poly(tetrafluoroethylene). *Langmuir* **18,** 6373–6380 (2002).
